# Synergistic Cytotoxicity of Permethrin and *N*,*N*‐Diethyl‐Meta‐Toluamide on Sinonasal Epithelial Cells

**DOI:** 10.1002/oto2.70145

**Published:** 2025-07-07

**Authors:** Jivianne T. Lee, Saroj K. Basak, Hong‐Ho Yang, Kimberly A. Sullivan, Tom Maxim, Daniel S. Shin, Nancy Klimas, Eri S. Srivatsan

**Affiliations:** ^1^ Department of Head and Neck Surgery David Geffen School of Medicine at University of California, Los Angeles Los Angeles California USA; ^2^ Department of Surgery Greater Los Angeles Veterans Administration Healthcare System, Los Angeles Los Angeles California USA; ^3^ Department of Otolaryngology–Head and Neck Surgery Stanford University School of Medicine Stanford California USA; ^4^ Department of Environmental Health Boston University, School of Public Health Boston Massachusetts USA; ^5^ Department of Clinical Immunology, Dr Kiran Patel College of Osteopathic Medicine Nova Southeastern University Miami Florida USA; ^6^ Miami Veterans Affairs Medical Center Geriatric Research Education and Clinical Center Miami Florida USA; ^7^ Department of Surgery David Geffen School of Medicine at UCLA Los Angeles California USA

**Keywords:** DEET, *N*,*N*‐diethyl‐meta‐toluamide, permethrin, pesticide, sinonasal epithelia

## Abstract

*N*,*N*‐Diethyl‐meta‐toluamide (DEET) and permethrin are pesticides commonly used in combination due to their synergistic insecticidal and repellent properties. This study investigates whether simultaneous exposure to these compounds elicits synergistic cytotoxicity in sinonasal epithelial cells (SNECs). Ethmoid sinus mucosal specimens were procured from eight patients during endoscopic sinus surgery. SNECs were expanded on culture plates and exposed to various concentrations of DEET and permethrin (0‐5 mM), individually and concurrently, for up to 156 hours. Experiments were replicated in triplets, and cell viability was recorded every 2 hours using *Incucyte* real‐time cell imaging system. Synergy score was calculated on the basis of the Loewe additivity synergy model. DEET and permethrin exhibited synergistic cytotoxicity across all eight tissues, albeit with variations in onset and magnitude. In conclusion, the concurrent exposure of DEET and permethrin can lead to synergistic cytotoxicity in sinonasal epithelia.

Pesticides are widely used in agricultural, industrial, and military settings. Despite their utility in pest control, growing concerns have arisen regarding their potential adverse effects on human health.^1^ For instance, pesticide exposure has been associated with upper respiratory conditions such as allergic rhinitis and chronic rhinosinusitis.^2^, ^3^, ^4^

The effects of common ingredients, permethrin and *N*,*N*‐diethyl‐meta‐toluamide (DEET), on sinonasal epithelial cells (SNECs) were studied in in vitro exposure experiments. These investigations revealed the cytotoxic effects of both ingredients on SNECs, with exposed specimens exhibiting cell death, morphological changes, and oxidative stress in a dose‐response fashion.^5^, ^6^

While understanding the cytotoxic effects of individual pesticide components is important, real‐world scenarios often involve simultaneous exposure to multiple ingredients. We present the first investigation into the presence of synergy between DEET and permethrin's cytotoxicity on SNECs.

## Materials and Methods

### Study Design

SNECs from the ethmoid sinus were procured during endoscopic sinus surgery and subsequently exposed to various concentrations of DEET and permethrin. Cell viability was monitored using a cell growth imaging system. Study approval was obtained from the Greater Los Angeles Department of Veterans Affairs IRB.

### Cell Culture and Viability Experiments

SNEC culture system was established in our lab as previously described.^5^, ^6^, ^7^ The primary monolayer cell cultures of SNECs were used to maintain the homogeneity of the culture system. SNECs were cultured in 96‐well plates and exposed to varying concentrations of permethrin (0‐5 mM) and DEET (0‐5 mM), independently and concurrently, for 156 hours. For each tissue, one well was left unexposed to serve as a negative control. Dosing concentrations and exposure schedules were based on previously established protocols.^8^, ^9^, ^10^ Cell proliferation and density were monitored with real‐time kinetic data using the *Incucyte* real‐time live cell imaging system (Essen Bioscience) and phase contrast images.^5^, ^6^ The wells were scanned, and data were recorded every 2 hours during the exposure period. Cell density was normalized using the starting level, and data were presented as fold changes since initiation (hour 0) to specific time points during the assay.

### Synergy Calculation

Synergy is quantified by measuring the degree to which the observed combined effect deviates from the predicted effect according to the null model. To quantify the synergy between DEET and permethrin, we utilized the *SynergyFinder* software and applied the Loewe additivity model to derive a synergy score (*S*_Loewe_). A score of 0 indicates perfect Loewe additivity; a positive score indicates synergy; and a negative score indicates antagonism.^11^

## Results

**Figure 1:**
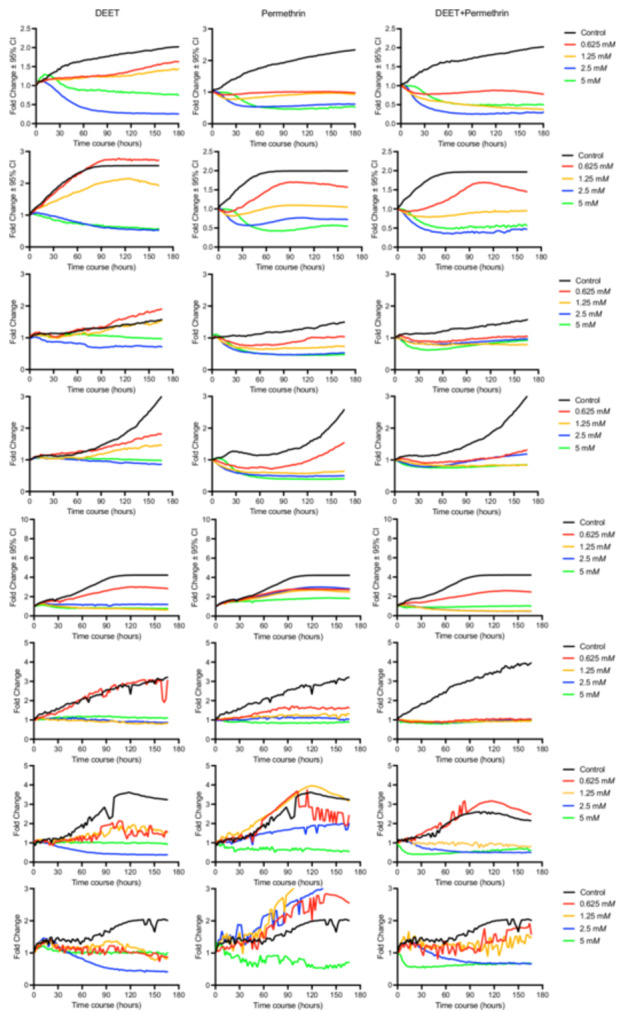
Cell viability of tissues 1 to 8 (top to bottom) when exposed to different concentrations of *N*,*N*‐diethyl‐meta‐toluamide (DEET) and permethrin, independently and concurrently.

Colorimetric assays of cell viability for SNECs from eight patients when exposed to various concentrations of DEET and permethrin are illustrated in Figure 1. Permethrin demonstrated greater cytotoxicity compared to DEET for tissues 1 to 6, but DEET demonstrated greater cytotoxicity compared to permethrin for tissues 7 to 8. By 2.5 mM, most SNECs were nonviable in both the DEET and permethrin experiments across most tissues. Across all tissues, co‐treatment with permethrin and DEET resulted in measurable synergy, with peak effects typically occurring between 96 and 144 hours. Table 1 details the onset and peak time points of synergy, the effective concentrations, and associated *S*_Loewe_ scores for each tissue.

**Table 1:** Time‐ and Dose‐Dependent Synergistic Effects of Permethrin and *N*,*N*‐Diethyl‐Meta‐Toluamide (DEET) Across Tissues

		Peak synergy		
Tissue	Synergy onset, h	Hour	Dose (permethrin + DEET), μM	Loewe synergy score (95% CI)
1	144	144	2.5 + 1.25	4.15 (1.84‐7.26)
2	48	96	1.25 + 0.625	19.87 (16.45‐23.61)
3	96	144	0.625 + 1.25	15.18 (3.88‐30.38)
4	144	144	0.625 + 0.625	6.42 (3.60‐18.00)
5	48	48	0.625 + 1.25	10.11 (8.86‐12.46)
6	48	96	0.625 + 0.625	24.65 (12.18‐36.30)
7	48	96	0.625 + 1.25	47.68 (29.57‐62.15)
8	48	96	0.625 + 0.625	47.41 (26.76‐66.98)

## Discussion

In this study, we exposed SNECs from the ethmoid sinuses of eight patients with DEET and permethrin. Significant synergy in cytotoxicity was observed across all tissues, although the onset and magnitude varied depending on the timepoints and concentrations.

Although the upper airway is readily exposed to sprayed pesticides, few studies to date have explored the toxicity of DEET and permethrin in sinonasal tissues. In a 2021 study involving three subjects, exposure of SNECs to DEET resulted in a dose‐dependent reduction in cell viability and morphological changes suggestive of cellular necrosis.^5^ Similarly, a 2022 study on permethrin demonstrated that increasing concentrations of in vitro exposure led to reduced viability and increased production of reactive oxygen species.^6^

Concurrent application of DEET on the exposed skin and permethrin on the clothing has been recommended for optimal protection against insects since DEET is vapor‐active and permethrin does not evaporate.^12^ However, our findings revealed significant synergistic cytotoxicity when SNECs were simultaneously exposed to DEET and permethrin. As the nasal cavity serves as the entry point for inhaled pollutants, the activity of cytochrome P‐450 (CYP450) is notably higher than that in the liver.^13^ As CYP450 is a vital enzyme in the metabolism of permethrin, we hypothesize that co‐administration with DEET could expedite the depletion of CYP450, thereby heightening cytotoxicity.^14^ Although our results do not establish a direct clinical link, prior epidemiologic evidence has connected pesticide exposure to chronic rhinosinusitis.^4^ Thus, our findings underscore the need for additional investigation into exposure thresholds and clinical relevance.

Indeed, it was previously contended that synergistic neurotoxicity resulting from the combination of DEET and permethrin would only manifest at very high concentrations.^12^ However, our experiments utilized concentrations significantly lower than the reported neurotoxicity‐inducing concentrations.^5^, ^6^ Although limited data exist on typical inhaled concentrations of pesticides, the concentrations used in our study fall within the range used in prior in vitro studies on sinonasal and immune cells.^6^, ^8^ We believe that any compound capable of producing the degree of synergistic cytotoxicity observed here merits further scrutiny. Future work is needed to address the lack of in vivo data quantifying sinonasal exposure concentrations.

## Conclusion

DEET and permethrin demonstrated in vitro synergistic cytotoxicity to sinonasal epithelial tissues.

## Author Contributions

**Jivianne T. Lee**, conceptualization, study design, writing—revision. **Saroj K. Basak**, data collection, study design, writing—revision. **Hong‐Ho Yang**, statistical analysis, writing—original draft, writing—revision. **Kimberly A. Sullivan**, conceptualization, study design, writing—revision. **Tom Maxim**, data collection, study design, writing—revision. **Daniel S. Shin**, data analyses, writing—revision. **Nancy Klimas**, conceptualization, study design, writing—revision. **Eri S. Srivatsan**, conceptualization, study design, writing—revision.

## Disclosures

### Competing interests

The authors of this study have no conflict of interest to declare.

### Funding source

The study was funded by the Department of Defense Grant W81XWH‐21‐2‐0048.
